# Identification of Aptamer-Binding Sites in Hepatitis C Virus Envelope Glycoprotein E2

**Published:** 2015-01

**Authors:** Fan Chen, Si-Chong Chen, Jing Zhou, Zhi-De Chen, Fang Chen

**Affiliations:** 1 Department of Biochemistry and Molecular Biology, Life Sciences School of Hubei University, Wuhan, China;; 2 Evolution and Ecology Research Centre, School of Biological, Earth and Environmental Sciences, The University of New South Wales, Sydney, Australia;; 3 Clinical laboratory, Wuhan Tuberculosis Dispensary, Wuhan Health Bureau, Wuhan, China;; 4 Department of Biochemistry and Molecular Biology, School of Basic Medical Science, Wuhan University, Wuhan, China

**Keywords:** Hepatitis C virus, Glycoprotein E2, Aptamer, Binding site

## Abstract

Hepatitis C Virus (HCV) encodes two envelope glycoproteins, E1 and E2. Our previous work selected a specific aptamer ZE2, which could bind to E2 with high affinity, with a great potential for developing new molecular probes as an early diagnostic reagents or therapeutic drugs targeting HCV. In this study, the binding sites between E2 and aptamer ZE2 were further explored. E2 was truncated to 15 peptides (P1 to P15) and these peptides were used to detect the affinity with ZE2 by ELISA respectively. The peptide with high affinity was then further truncated, detected and compared with six kinds of HCV genotypes. The basic amino acid in 500 aa bound to ZE2 with high affinity, while acidic amino acid in 501 aa reduced the reaction between E2 and ZE2. The results showed the 500 aa and 501 aa of E2 were the key sites that bound to ZE2.

## Introduction

Hepatitis C Virus (HCV) is a major cause of chronic liver diseases worldwide. It is estimated that 3% of the world’s population have been infected by HCV, and the infection usually results in liver cirrhosis, in some cases, hepatocarcinoma.^1^ Recent reports have shown more HCV recurrence and decreased survival in patients with chronic hepatitis C.^2^ It is therefore vital to develop new reagents of diagnosis and therapies for these infections due to the high prevalence of HCV, Human Immunodeficiency Virus (HIV), and Hepatitis B Virus (HBV) co-infection.

HCV encodes two envelope glycoproteins, E1 and E2, which play crucial roles in the initiation of infection by mediating the interaction between the virus and the host cell membrane.^3^^,^^4^ While E2 is thought to initiate viral attachment, E1 may be involved in virus-cell membrane fusion.^4^^,^^5^ E2 has been proposed to be responsible for recognizing and binding to cellular receptors.^6^ CD81 has been identified as a critical co-receptor for HCV particle entry.^7^

Aptamers could inhibit the virus at the stage of viral entry by blocking the interactions between the viral envelope glycoprotein and the cellular receptor.^8^ Our previous work reported a DNA aptamer ZE2 could block HCV E2 from binding the CD81 receptor and the infection to human hepatocytes. However, such abilities in six HCV genotypes are different. The binding sites of E2 were further explored in this paper to clarify the mechanism of E2 in the infection of hepatocytes.

## Materials and Methods

*Cell Lines and Aptamers*

Ectopically expressing HCV-E2 envelope glycoprotein CT26 cell lines (E2-CT26 cells) and parental CT26 cells (mock cells) were established by G418 selection^9^ (CT26 was purchased from The Culture Collection of Wuhan University), and cultured in RPMI 1640 medium with 10% (v/v) fetal bovine serum (Gibco/Invitrogen). Rabbit anti-E2 polyclonal antibody was prepared as in our previous publication. Aptamer ZE2 and its mutated aptamer ZE2-mut, were synthesized (Sangon Biotech Co., Ltd., Shanghai, China) as previously described.^10^

*Aptamer* *Bound with E2-Expressing Cells*

The method of aptamer ZE2 bound with E2-CT26 or CT26 cells was as reported previously. In brief, FITC labeled aptamer incubated with cells (E2-CT26/CT26) in six well plates at 4ºC overnight. Each well had a cover slide, after incubation, washed with PBS and fixed with paraformaldehyde. Then, the fixed cells were stained with propidium iodide and imaged with a confocal fluorescence microscope. These tasks were carried out from 2011 to 2012 at the School of Basic Medical Science, Wuhan University, China.

*E2 Peptides Synthesis and ELASA*

To further identify ZE2 peptide-binding site on E2, 15 peptide fragments (from P1 to P15), which were based on the genotype 1a E2 amino acid sequence and secondary structure, were synthesized (HD Biosciences Co. Ltd. Shanghai, China) and each E2 fragment (from P1 to P15) were used to coat ELISA plates and incubated with biotin-labeled ZE2. The biotin-ZE2-bound E2 fragments in the ELISA plate were revealed by the HRP labeled streptavidin. For the analysis of the ZE2 binding site on E2 protein, the ELISA plates were coated with 0.3 nM at 4℃ for 7 h. After the plates were blocked with 2% BSA and washed with PBS, 250 nM biotin-labeled ZE2 was added to each well. To locate the key amino acid of E2 involved in binding to ZE2, peptides fragment with the strongest affinity to ZE2 was truncated again, the same steps as those described above were then followed. ZE2-mut was used as control to analyze the differences of affinity.

*Statistical Analysis*

Experimental data were analyzed by ANOVA or an unpaired Student’s t test. P<0.05 was considered statistically significant. 

## Results

*Observation of the Aptamer Binding to E2-expressing Cells by Confocal Microscopy*

**Figure 1:**
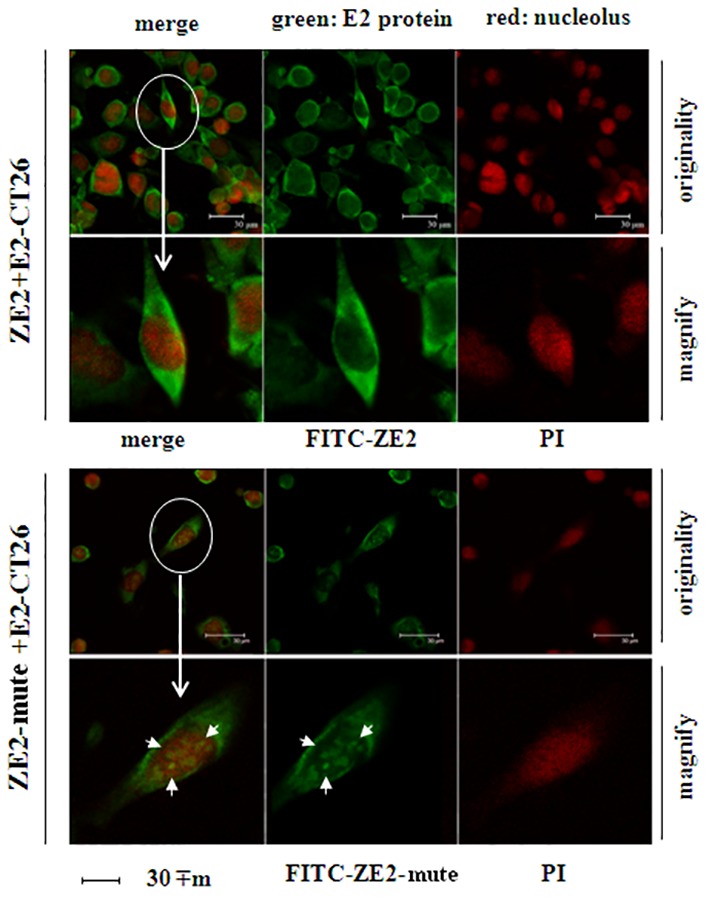
Fluorescence microscope imaging of E2-expressing cells with FITC-ZE2 and FITC-ZE2-mut. A) FITC-ZE2 bound to the surface of E2-CT26 cells by confocal immunofluorescence microscopy. B) FITC-ZE2-mut was intracellular of the E2-CT26 cells by confocal immunofluorescence microscopy. Each test has repeated six times.

The E2-expressing cells, E2-CT26, specifically bound FITC-ZE2 aptamer under the fluorescence microscope, indicating the affinity between synthesized aptamers and E2. The fluorescence intensities of the binding affinities between FITC-ZE2 and E2-expressing cells were higher than that between FITC-ZE2-mut and E2-expressing cells. Interestingly, we further observed ZE2-mut could enter into the cells, while ZE2 could only bind to the cells on the surface (figure 1). Previous studies reported that the synthesized aptamer ZE2 had similar function^10^ and the difference of ZE2 and ZE2-mut binding to E2-expressing cells was closely related to their structures.

**Table 1:** Peptides sequences were synthesized (P1 to P15).

**Synthetic peptides **	**E2 peptides sequences **
P1 (384-403)	ETHVTGGSAGRTTAGLVGLL
P2 (404-424)	TPGAKQNIQLIDTNGSWHINS
P3 (425-437)	TALNCNESLNTGW
P4 (438-455)	LAGLFYQHKFNSSGCPER
P5 (456-475)	LASCRRLTNFAQGWGPISYA
P6 (476-488)	NGSGLDERPYCWH
P7 (489-508)	**YPPRPCGIVPAKSVCGPVYC**
P8 (509-529)	FTPSPVVVRTTDRSGAPTYSW
P9 (530-549)	GANDTDVFVLNNTRPPLGNW
P10 (550-572)	FGCTWMNSTGFTKVCGAPPCVIG
P11 (573-592)	GVGNNTLLCPTDCFRKHPEA
P12 (593-609)	TYSRCGSGPWITPRCMV
P13 (610-629)	DYPYRLWHYPCTINYTIFKV
P14 (630-642)	RMYVGGVEHRLEA
P15 (643-658)	ACNWTRGERCDLEDRD

*Identification of the Binding Site of E2*

**Figure 2:**
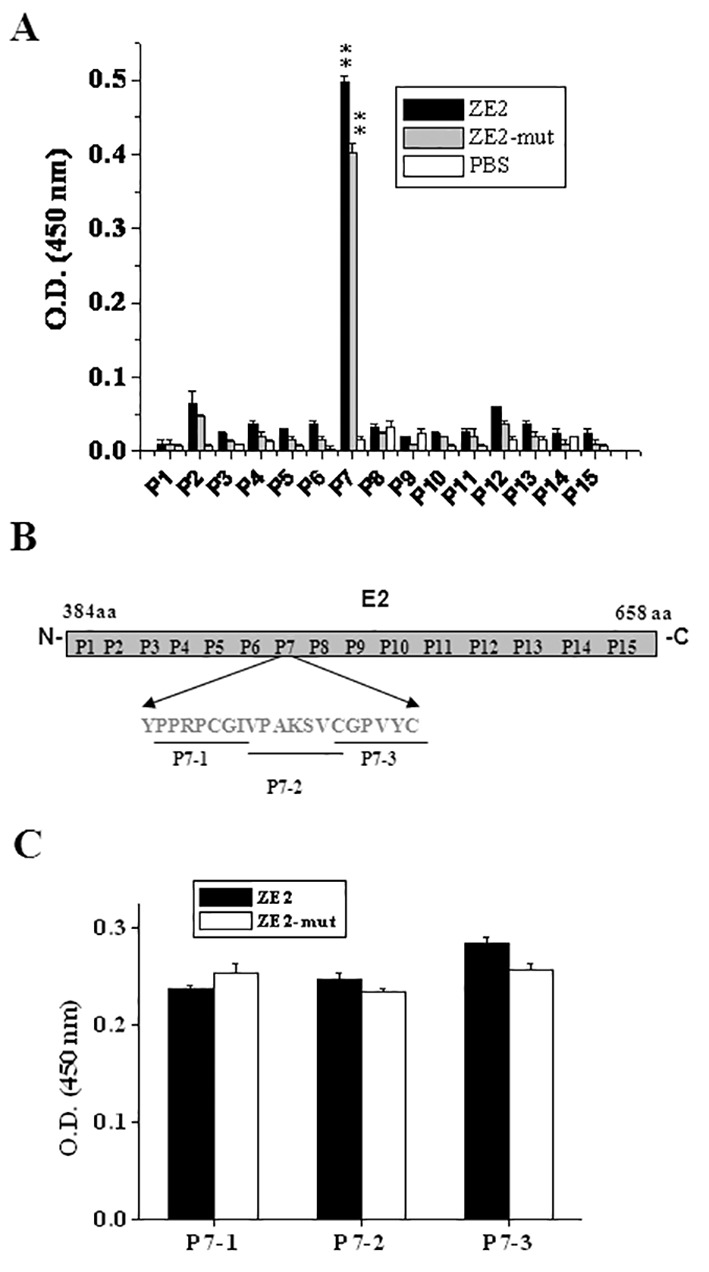
Identification of the ZE2-binding site of HCV E2 envelope glycoprotein. A) The binding affinity between aptamers and E2 fragment (from P1 to P15) was detected by indirect ELISA. B) Diagram of the 15 E2 peptide fragments (from P1 to P15) that spanned different regions of E2, and the P7 fragment was truncated to P7-1, P7-2, and P7-3. C) The binding affinity was detected between aptamers and three peptides of P7. Each test has been repeated three times. (**P<0.01)

To explore the ZE2 binding site on E2, fifteen peptide fragments were synthesized, which were based on the genotype 1a E2 amino acid sequence and secondary structure (table 1). Those fifteen fragments were used to coat ELISA plates and incubated with biotin-labeled ZE2. The binding affinity was detected by ELISA, as described earlier. Among those fifteen fragments, the results suggest that ZE2 bound most strongly to the E2-P7 fragment sequence, while ZE2-mut showed slightly lower binding affinities than ZE2 (figure 2). The binding affinity between ZE2 and P7 was 2- to 20-fold higher compared with those between ZE2 and the other 14 peptides (P=0.00). Since ZE2 bound most strongly to the E2-P7 fragment sequence, we further truncated E2-P7 to three smaller fragments: P7-1, P7-2, and P7-3 (figure 2). The indirect ELISAs were used to detect the affinity between the aptamer and peptides (P7-1, P7-2, and P7-3). The results indicated that there is no significant difference of binding to peptides between ZE2 and ZE2-mut.

*Analysis of the Binding Site between the Aptamers and the Protein*

We compared the recognized E2 sequences (the P7 fragment sequences) in different genotypes of HCV E2 by GenBank and found several critical amino acids in the E2-P7 fragment (table 2). Most amino acids of the P7 fragment were conserved, but two regions of amino acids that have a positive charge (500 aa and 501 aa) were closely related to the combination of aptamers. Moreover, when these sites were alkalescence amino acid, the P7 fragment showed higher affinity with aptamers than those neutral amino acids or acidic amino acids, suggesting the specific recognition ability of the aptamer to these sites of E2.

**Table 2:** Comparison of E2-P7 fragment sequences in several HCV E2 genotypes

**HCV genotype**	**Isolated strain**	**HCV E2 P7 peptides sequences (488-508 aa)**	**GenBank** ** Acc. No.**
1a	H77	H488YPPRPCGIVPA**KS**VCGPVYC508	AAB67038
1b	HC-J4/91	H488YAPRPCGXVPA**SQ**VCGPVYC508	AAC15725
2a	JFH1	H488YPPKPCGVVPA**RS**VCGPVYC508	BAB32872
3a	K3a/650	H488YAPRPCGIVPA**LN**VCGPVY C508	BAA06044
4a	ED43	H488YAPRPCGIVPA**SS**VCGPVY C508	CAA72338
5a	EUH1480	H488YPPRPCGVVPA**RD**VCGPVY C508	CAA73640
6a	6a33	H488YAPRPCDVVPA**ST**VCGPVY C508	AAW56714

K: Lysine; R: Arginine; N: Asnaragine; Q: Glutamine; D: Aspartic acid; S: Serine; T: Threonine; L: Leucine

## Discussion

The most common genotypes of HCV in China are subtypes 1b and 2a, followed by 1a or 6.^11^ Our previous study found that ZE2 could bind specifically to HCV-E2 (genotype 2a) by cell surface-systematic evolution of ligands by exponential enrichment (CS-SELEX). The binding affinities of ZE2 with diverse genotypes were different, and the affinities of aptamer ZE2 with genotypes 1a, 1b and 2a of E2 were higher than other genotypes.

This study furthermore demonstrated that ZE2 could capture E2-expressing cells, and both ZE2 and ZE2-mut could bind to E2-P7 fragment. Although lower fluorescence intensity was observed when using the FITC-ZE2-mut bound to E2-express cells than doing the FITC-ZE2, it was found that ZE2-mut could enter into intracellular while ZE2 only bound to the surface of E2-express cells (figure 1). We assume that the mutation of two bases in the terminal loop of a stem-loop structure of ZE2 changed the binding property, which made the cell surface combining (ZE2) turn to cells intake (ZE2-mut). The result indicated E2 was not only initiate viral attachment, but also may be involved in the virus-cell membrane fusion.

ZE2 binds to P7 fragment of E2 stronger than to other fragments (figure 2), and amino acids 500 and 501 are closely related to the combination of the aptamer. When these sites are mutated to basic amino acids, the P7 fragment shows higher affinity with the aptamer, making ZE2 bind to genotypes 2a, 1a, and 1b of E2 stronger than to other genotypes. Even though the P7 fragment of 1b does not consist of basic amino acids in 500 aa and 501 aa; it still has a high affinity with ZE2. Further, the 496 amino (X) possibly plays an important role. It may be a basic amino acid or provide special space structure to recognize ZE2. By studying all genotypes P7 fragment of E2, we found its specific characters: there are many prolines in P7 fragment (15%~20%), making the peptide unique tertiary structure; the basic amino acid could strengthen the affinity with ZE2 while acidic amino acid could weaken this combination. For example, although the 500 aa in genotype 5a of E2 is arginine, the aspartic acid of 501 aa makes less affinity with ZE2.

Several reports showed that HCV cell entry is a multi-step process.^12^ Some conserved residues in HCV E2 for CD81 binding were identified and three putative CD81 interacted sites on HCV E2 have also been previously identified.^5^ ZE2 is partially capable to block HCV E2 from binding to the CD81 receptor and subsequent viral entry and infection of human hepatocytes. CD81 binds most tightly with the P7 fragment (amino acids 489 to 508) of E2 as well.^10^ These data demonstrate that ZE2 and CD81 share similar binding sites on the P7 fragment of E2, but it needs further investigation

Although two binding sites were found in this study, it needs more proof to demonstrate the interaction between these binding sites and NK2. E2 was truncated to fifteen fragments, which might destroy its space-conformation, consequently, missed the right binding site. Therefore, it is important to detect the binding site of E2 in the condition of keeping its space-conformation. 

## Conclusion

This study demonstrates that the binding site of ZE2 binding with E2 is P7 fragment. 500 aa and 501 aa on P7 fragment of E2 are the critical amino acids that bind to ZE2. Moreover, P7 fragment of E2 not only recognize ZE2 and ZE2-mut, but also decide the mode of action, outside binding or cells intake, which needs further researches. 

## References

[1] Memon MI, Memon MA (2002). Hepatitis C: an epidemiological review. J Viral Hepatitis.

[2] Hsu CS, Liu CJ, Liu CH, Wang CC, Chen CL, Lai MY (2008). High hepatitis C viral load is associated with insulin resistance in patients with chronic hepatitis C. Liver Int.

[3] Lindenbach BD, Rice CM (2003). Molecular biology of flaviviridae. Adv Virus Res.

[4] Flint M, McKeating JA (2000). The role of the hepatitis C virus glycoproteins in infection. Rev Med Virol.

[5] Rothwangl KB, Manicassamy B, Uprichard SL, Rong L (2008). Dissecting the role of putative CD81 binding regions of E2 in mediating HCV entry: putative CD81 binding region 1 is not involved in CD81 binding. Virol J.

[6] Cocquerel L, Voisset C, Dubuisson J (2006). Hepatitis C virus entry: potential receptors and their biological functions. J Gen Virol.

[7] Meuleman P, Hesselgesser J, Paulson M, Vanwolleghem T, Desombere I, Reiser H (2008). Anti-CD81 antibodies can prevent a hepatitis C virus infection in vivo. Hepatology.

[8] Bunka DH, Stockley PG (2006). Aptamers come of age - at last. Nat Rev Microbiol.

[9] Li P, Wan Q, Feng Y, Liu M, Wu J, Chen X (2007). Engineering of N-glycosylation of Hepatitis C Virus Envelope Protein E2 Enhances T cell responses for DNA immunization. Vaccine.

[10] Chen F, Hu Y, Li D, Chen H, Zhang XL (2009). CS-SELEX Generates High-Affinity ssDNA Aptamers as Molecular Probes for Hepatitis C Virus Envelope Glycoprotein E2. PLoS One.

[11] Lu L, Nakano T, He Y, Fu Y, Hagedorn CH, Robertson BH (2005). Hepatitis C virus genotype distribution in China: predominance of closely related subtype 1b isolates and existence of new genotype 6 variants. J Med Virol.

[12] Pietschmann T ( 2009). Virology: Final entry key for hepatitis C. Nature.

